# Enhancing the usability and performance of structured association mapping algorithms using automation, parallelization, and visualization in the GenAMap software system

**DOI:** 10.1186/1471-2156-13-24

**Published:** 2012-04-03

**Authors:** Ross E Curtis, Anuj Goyal, Eric P Xing

**Affiliations:** 1 Joint Carnegie Mellon - University of Pittsburgh PhD Program in Computational Biology, Carnegie Mellon University, Pittsburgh, PA 15213, USA; 2 Lane Center for Computational Biology, Carnegie Mellon University, Pittsburgh, PA 15213, USA; 3 Language Technologies Institute, Carnegie Mellon University, Pittsburgh, PA 15213, USA; 4 Machine Learning Department, Carnegie Mellon University, Pittsburgh, PA 15213, USA

## Abstract

**Background:**

Structured association mapping is proving to be a powerful strategy to find genetic polymorphisms associated with disease. However, these algorithms are often distributed as command line implementations that require expertise and effort to customize and put into practice. Because of the difficulty required to use these cutting-edge techniques, geneticists often revert to simpler, less powerful methods.

**Results:**

To make structured association mapping more accessible to geneticists, we have developed an automatic processing system called Auto-SAM. Auto-SAM enables geneticists to run structured association mapping algorithms automatically, using parallelization. Auto-SAM includes algorithms to discover gene-networks and find population structure. Auto-SAM can also run popular association mapping algorithms, in addition to five structured association mapping algorithms.

**Conclusions:**

Auto-SAM is available through GenAMap, a front-end desktop visualization tool. GenAMap and Auto-SAM are implemented in JAVA; binaries for GenAMap can be downloaded from http://sailing.cs.cmu.edu/genamap.

## Background

High-throughput technology has resulted in an explosion of biological data including gene expression and SNP data for a growing number of organisms. In order to understand the biological/medical implications and mechanistic insights behind such ever-growing amounts of data, biologists have relied on advances in statistical learning and inference technology to give them the tools they need to elucidate relationships between genes, genomic mutations, and phenotypic traits. The need for powerful analytic tools is especially pertinent in genetic association mapping. In a genetic association mapping study, millions of genomic markers, usually single-nucleotide polymorphisms (SNPs), are collected for a cohort of patients. In addition to the vast amount of genomic data, gene expression data for thousands of genes and trait measurement data for hundreds of clinical traits are also collected. The genetics analyst must explore this vast amount of complex, structured data to find SNPs that are associated with genes or traits of interest. Many successful association studies have been performed and provided insight into a variety of human diseases [1-4].

Despite the success of association mapping in uncovering SNPs associated with disease, traditional genome wide association studies (GWAS) that look for associations between the genome and phenotypic traits are limited in their ability to identify and explain causal SNPs [1]. For example, the results of many GWAS point to SNPs that account for only a small fraction of the disease heritability [5], or point to SNPs that do not affect protein sequence, suggesting involvement in some unknown regulatory pathway [6]. Steps to incorporate other data, such as genome-wide expression data, into association analysis have shown some promise [7]; however, researchers often rely on simple, single-SNP to single-trait association tests to find pairwise SNP-gene associations, even in studies with large sets of correlated genes or traits [8]. Thus, despite the incorporation of additional data, association mapping studies are still hampered by the use of methods that ignore structures (and thus information) inherent in the data.

Recent advancements in machine learning have led to a new generation of GWA technologies, termed *structured association mapping *algorithms, which can leverage inherent structure present in the genome and phenome when performing association mapping analysis. For example, GFlasso [9] and TreeLasso [10] algorithms both consider the presence of complex correlation structures among individual traits that constitute the so-called *intermediate phenotypes *(measurements of dependent clinical traits that describe a symptom, or genes that constitute a functional module) in the discovery of associated SNPs. Other algorithms, such as MPGL [11] or AMTL [12], leverage genomic structure, such as population structure and known genomic features, to identify associations. Indeed, structured association mapping is proving to be a powerful statistical tool with the potential to enhance the discovery of weaker signals while eliminating false positives [9].

While the promise of structured association mapping has been shown through technical papers and in mathematical conferences, its application to genetics study has been significantly slower. The traditional presentation and deployment of methodological advances in machine learning and statistics fields hinders their acceptance and use by biologists. For example, structured association mapping algorithms are generally made available as crude, command-line implementations (if they are made available at all). Thus, in order for a geneticist to use a structured association mapping algorithm in a GWAS analysis, they must download a rough MATLAB implementation of the algorithm and customize the code to fit their study. Furthermore, many of these algorithms have been scaled and tested only on small, simulated datasets, and while they can be potentially extended to larger datasets, the way to do so is not obvious to those not specialized in machine learning or familiar with the mathematical details. Due to the amount of specialization required to run the algorithms, the algorithms stay on the shelf, despite their potential to aid in genetics analysis.

In this paper, we propose a new strategy and a user-oriented fully automated software system for the deployment of new machine learning algorithms with the purpose to make them accessible to geneticists. We believe that the wide-spread acceptance of such an approach could potentially accelerate biological discovery by facilitating the incorporation of cutting-edge machine learning techniques. More specifically, we have developed a software system, which we call Auto-SAM, which automates the execution of five structured association mapping algorithms, four association mapping algorithms, and four structure-finding algorithms (Table 1). In contrast to the general strategy of posting a raw implementation on the web, we systematically develop each algorithm so it will automatically run in a distributed parallel-computing environment. Thus, little technical specialization is required for a genetics analyst to run the algorithms.

**Table 1:** Algorithms available to run in Auto-SAM

Algorithm	Type	Input	Output	Mouse run Time (traits)	Mouse run time (genes)	Yeast run time	Automated steps
**GFlasso **[13]	SAM	G, P, Ep	G-P association	0 05:05:50	1 16:17:45	2 06:43:56	17

**MPGL **[11]	SAM	G, P, Pop	G-P association	2 09:07:47	-	3 11:09:46	3

**TreeLasso **[10]	SAM	G, P, Ep	G-P association	0 01:12:03	0 12:53:52	0 05:08:42	15

**AMTL **[12]	SAM	G, P, Fg	G-P association	-	-	1 20:35:47	8

**gGFlasso **[14]	SAM	T, Et, P, Ep, G/T assoc	T-P association	N/A	0 01:54:04	N/A	19

**Wald Test **[15]	AM	G, P	G-P association	0 00:23:29	0 09:51:23	0 00:54:04	5

**Wilcoxon Sum-rank test **[8]	AM	G, P	G-P association	0 00:10:21	0 01:05:51	0 00:14:32	4

**Lasso **[16]	AM	G, P	G-P association	0 00:59:21	0 08:22:42	0 04:07:53	6

**association by population **[17]	AM	G, P, Pop	G-P association	0 00:57:24	2 19:02:46	1 03:00:44	5

**Correlation**	network	P	Ep	0 00:01:50	0 00:06:05	0 00:09:29	3

**Glasso **[18]	network	P	Ep	0 00:07:31	0 01:19:37	0 01:51:24	10

**Scale-free network **[19]	network	P	Ep	0 00:03:11	0 00:41:23	0 00:41:08	6

**Hierarchical clustering**	tree	P	tree	0 00:43:32	0 01:27:32	0 01:05:04	3

**Structure **[20]	population	G	Pop	0 13:30:32	0 13:30:32	0 00:41:54	4

**Gene module discovery **[8]	Network analysis	Ep	phenotype clusters	N/A	0 12:29:24	0 01:03:22	4

We present a list of all algorithms available to run through the Auto-SAM system and GenAMap. We group the algorithms by type: SAM (structured association mapping), AM (association mapping), network generation, tree generation, population determination, and network analysis. The input for each algorithm can be G (genotype), P (phenotype), T (gene expression data), Ep (edges for the phenotype), Fg (features of the genotype), Et (edges for the gene expression values), and G/T (genome/transcriptome) associations. Times are represented as D HH:MM:SS where *D *day, *H *hour, *M *minute, and *S *second.

We have integrated Auto-SAM with GenAMap, which is a visualization tool for structured association mapping [21,22]. In GenAMap, we have used cutting-edge visualization research to design visualization schemes specific to the problems faced in structured association mapping analysis. By integrating Auto-SAM with GenAMap, we provide an environment where genetics analysts can run algorithms automatically and also use powerful visualizations to explore and interpret structure and association results.

It might be argued that one common approach of deploying new algorithms via CRAN-R libraries [23] is similar to the strategy we propose here. (For examples see glasso [18] or bioconductor [24]). However, our approach differs in three significant ways: 1) by running algorithms in parallel on a distributed system with access to a cluster-computing system, Auto-SAM is able to handle larger datasets than would be possible with an R library; 2) through the use of a database, analyses are made available to entire teams of analysts; 3) the integration of Auto-SAM with GenAMap provides state-of-the-art visual analytic tools that enable the analyst to explore and analyze the data and results, including links to external databases and integration with gene-ontology resources.

We show the validity of our approach by reporting the running times of Auto-SAM's algorithms on two publically available association mapping datasets, noting that the time and specialization needed to run a similar analysis without Auto-SAM is several orders of magnitude greater. We demonstrate the complexity of our design of how we run each algorithm through a discussion of the GFlasso integration with Auto-SAM. Through this discussion we show that by using Auto-SAM, we are able to run GFlasso on larger datasets than would be possible with only the source code. Auto-SAM is available through GenAMap, which can be downloaded for non-commercial use at http://sailing.cs.cmu.edu/genamap.

## Implementation

### Overview of the software

**Figure 1:**
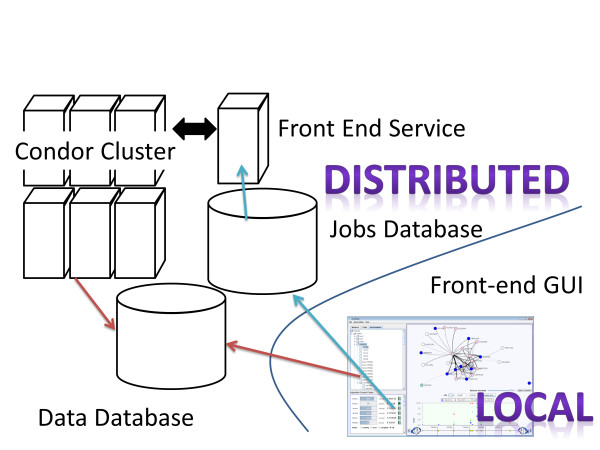
**Software design of the Auto-SAM system**. Locally, through a front-end GUI, the user uploads data to the data database. The GUI also communicates with the jobs database, submitting job requests that use the loaded data. A service running on the distributed Auto-SAM system continuously monitors the jobs database, which spawns and monitoring jobs as they run through the condor cluster.

We have designed and implemented a software system (Auto-SAM) to automatically run structured association mapping algorithms. Auto-SAM runs on a distributed system, which communicates with a front-end GUI run locally on the analyst's machine (Figure 1). There are two databases in Auto-SAM. The *data database *holds data: SNP data, gene expression data, association mapping results, etc. The *jobs database *stores information about each job request from an analyst. Once an analyst has uploaded data into the data database, he or she can request to run a job through the jobs database. A service running on the distributed machine then runs the job on a cluster, monitors its progress, and updates the jobs database so the front-end GUI can notify the analyst of its progress.

### Data management

All data and results that are used by Auto-SAM are stored securely in the data database. We have implemented the data database using MySQL. An analyst would use a front-end GUI like GenAMap to upload data into the database. Auto-SAM organizes all data by team, where each team is made up of one or more analysts that all have access to the same data. Analysts can only access data from their own team. Each team's data are organized by project, and each project is made up of marker, trait, structure, and association data.

Storing the data in a remote database allows for easy data sharing between all analysts on a team, and also provides the cluster with quick access to the data. Additionally, the storing of the data on a remote database allows the front-end GUI to query for data by-need to avoid the costly storing of all the data in a local machine's memory. Auto-SAM takes advantage of the fact that many results (such as gene-gene networks or SNP-gene associations) are sparse, and thus saves considerable space by only storing non-zero values. Auto-SAM is built so that it can be integrated with new, faster database technologies when the need arises.

### Algorithm automation and parallelization

Once the analyst has uploaded marker and trait data, he or she can run algorithms to find structure in the data using Auto-SAM. He can also run association mapping algorithms, structured association mapping algorithms, or other analysis algorithms. Most of the algorithms available to run in Auto-SAM are shown in Table 1. All algorithms are monitored by the Auto-SAM service, which runs continuously on the distributed system.

The Auto-SAM service monitors and updates the jobs database, which keeps track of each job as it goes through a series of steps to run the algorithm. Each available algorithm is defined in the jobs database, with a pointer to the executables needed to run each step in the algorithm. In order to run an algorithm, the front-end GUI requests a job of a specific type and specifies the datasets to be used. The service will then start the job from its first step. The service has only two functions: 1) monitor and update the jobs database, and 2) run and monitor jobs on the cluster. The Auto-SAM service is implemented in JAVA. Auto-SAM uses a 240 node computing-cluster that is running condor to execute all steps of each algorithm [25].

Each algorithm that Auto-SAM runs has a series of steps predefined, each with a specific executable. The steps are completed sequentially. All algorithms have a front-end step to download data from the database and prepare for subsequent steps. Once all steps have finished successfully, the back-end step inserts the results into the database. Thus, each processing pipeline only touches the database at the first and last steps. This allows for the quick and easy integration of new algorithms into Auto-SAM, without the integration of database management into the code.

**Figure 2:**
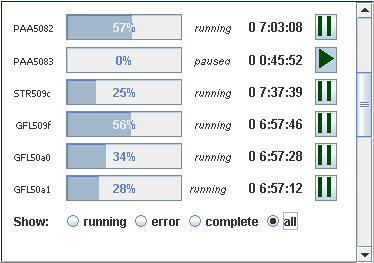
**Monitoring jobs in Auto-SAM**. We implemented a job-monitoring system that regularly checks the progress of each job in the database. Using this monitor, the analyst can follow each job's progress, request error information, and pause and kill jobs. This job monitor is integrated into GenAMap.

At the completion of all jobs in a step, the service checks to make sure that there were no errors and moves the job to the next step. If an error did occur, the job is stopped and the analyst must choose to restart it or kill it altogether. At any time an analyst can choose to pause or kill a job. We have implemented a front-end control panel in GenAMap that monitors the progress of each job, allowing the analyst to pause, restart, and remove jobs (Figure 2). By right-clicking on any of the labels in a job, the analyst can select to remove or restart a job, or to query for available error information.

The integration of an algorithm into Auto-SAM can be done by simply adding a front and back end, or through the complex compilation of several processing steps. We have used both of these strategies in designing and incorporating algorithms into Auto-SAM. For example, we have automated the Wald test [15] (from PLINK) for association between the genome and quantitative traits into Auto-SAM. In this case, the front-end step formats the data, the second step runs PLINK, and the final backend step inserts the results into the database. We used a similar strategy to incorporate *Structure *[20], a popular algorithm to find population structure, into Auto-SAM.

On the other hand, we have taken advantage of the cluster in our integration of other algorithms into GenAMap. For example, when running a lasso job using glmnet [16], Auto-SAM splits the dataset up into jobs the size of 250 traits and runs these each five times separately (using different data splits for a defined vector of the regularization parameter, *λ*). Upon completion of this step, the next step reads in the results from each of the runs to choose the best *λ *based on cross-5-validation error over all traits. Similarly, when Auto-SAM calculates a correlation network, it splits up the network into sections of 1000 traits and calculates the values for each section in parallel, allowing the job to run much faster. While parallelization using Auto-SAM allows analysts to run jobs much faster than they would run otherwise, the running time of each job is also affected by the number of jobs in the cluster and the number of threads accessing the database.

### Visualization front end

We have integrated Auto-SAM with GenAMap, a visualization tool that we have previously presented for structured association mapping [22]. GenAMap is a desktop visualization tool that enables genetics analysts to explore the structure of genes and traits while considering associations to the genome. Multiple coordinated views allow analysts to consider structure in the SNPs and traits simultaneously while considering the associations between the two data types.

The current implementation of GenAMap uses Auto-SAM to store the data it presents to analysts. GenAMap communicates with Auto-SAM via a web interface. GenAMap itself does not perform association analysis, but rather is designed for the visualization and exploration of association results. It is through Auto-SAM that analysts can run algorithms to find associations and structures in the data. However, analysts can by-pass Auto-SAM and upload network and association results from other tools to explore in GenAMap.

**Figure 3:**
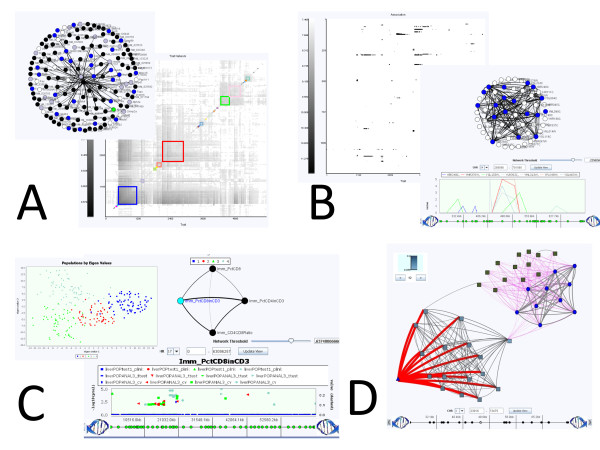
**An overview of GenAMap visualization tools**. GenAMap is a visualization tool for association mapping. We present a sampling of visualizations available in GenAMap: A) network analysis, B) association analysis, C) association-by-population analysis, D) three-way genome-transcriptome-phenome analysis.

GenAMap's visualization tools enable genetic network exploration, population structure analysis, and the exploration of association results. Analysts can directly link to information in outside resources such as UniProt [26] or dbSNP [27]. A selection of GenAMap's visualizations is highlighted in Figure 3, including gene network visualization, three-way association visualization, and association by population.

## Results

Auto-SAM automates five structured association algorithms (GFlasso, TreeLasso, AMTL, MPGL, and gGFlasso [14]), four structured association mapping algorithms (the Wald test, the Wilcoxon Sum-Rank test, the lasso, and a simple population analysis [17]), and five structure-generating algorithms (structure, correlation, hierarchical clustering, scale-free network construction [19], and glasso). As we incorporate each algorithm into Auto-SAM, we test each step on simulated and real datasets. We compare the results from Auto-SAM with the results from running each software piece outside of the automated processing system. Thus, we are confident that the results found by Auto-SAM are the same as if the code was run outside of the automatic processing environment.

To demonstrate Auto-SAM, we present the running times of each algorithm on two publically available datasets. The first dataset that we use is a yeast expression dataset [28] consisting of 1260 SNPs and 5637 gene expression measurements for 114 individual yeast strains. The second dataset is the NIH heterogenous stock mouse dataset [29]. We use the phenotypic traits and the gene expression measurements from the liver in our analysis of the stock mice. Thus, we have 12545 SNPs for 259 individual mice, which match up with the phenotype trait set of 158 clinical measurements and an expression dataset with 5965 gene expression measurements.

In Table 1, we list the running time from each algorithm, averaged over three independent runs, on the three publically available datasets. We also list the number of automated steps in Auto-SAM needed to complete each algorithm. To generate these results, we ran each algorithm in parallel in Auto-SAM simultaneously with up to ten other algorithms. We monitored the cluster to limit the amount of time when all compute nodes were busy, and we limited the number of threads hitting the database to eight at any one time. We believe that the times we report are representative of what an analyst might expect with a heavy load in Auto-SAM. We suggest that these running times are orders of magnitude faster than downloading the implementations, editing data into the correct formats, and piecing the structure and association algorithms together to form a processing pipeline.

In Table 1 we list the five structured association mapping algorithms we have integrated into Auto-SAM. Each of these algorithms has an available implementation online, but these implementations are not scalable to the size of the data in our three datasets. After loading these data into the Auto-SAM system, we were able to complete the algorithmic analysis for each of the five structured association mapping approaches in significantly less than a week's time. This time was all computational running time, and thus required very little input from us. In order to perform a similar analysis without the Auto-SAM system, we would have had to download the online implementation, preprocess our data so that it would run in a reasonable amount of time, and then run the analysis. The details of the integration of each of these algorithms into Auto-SAM are detailed online from http://sailing.cs.cmu.edu/genamap. Here, we present one example, the integration of GFlasso with Auto-SAM to demonstrate the complexity of the automated analysis that we undertake to scale these algorithms to larger datasets than is possible with current structured association mapping implementations.

### Implementation case study: GFlasso

Auto-SAM's parallel processing environment can be leveraged to increase the scalability and decrease the running time of GWA and structured association mapping algorithms. To demonstrate how this can be done, we use our integration of GFlasso into Auto-SAM as a case study. We show how we have increased the scalability of GFlasso by adding processing steps and by using the cluster in Auto-SAM. We give an overview of this processing pipeline in Table 2. For analysts to run GFlasso on their data without Auto-SAM, they would have to develop a similar process, writing their own code for each step. This example is presented to demonstrate the advantages of using Auto-SAM, to show how new algorithms can be added to Auto-SAM, and to enable developments using similar strategies in future algorithm deployment.

**Table 2:** GFlasso processing pipeline

**Step No**.	Description	Stage	No. Jobs using yeast data	Av. Time/Job on yeast data Hours:min:sec	Actual time from start to finish	Time saved via parallelization
**1**	Lasso stage 1	Preprocessing SNPs	23	00:00:25	00:02:59	00:06:36

**2**	Lasso validation error	Preprocessing SNPs	1	00:02:14	00:02:14	00:00:00

**3**	Lasso stage 2	Preprocessing SNPs	23	00:01:16	00:03:40	00:25:34

**4**	Lasso validation error	Preprocessing SNPs	1	00:00:39	00:00:39	00:00:00

**5**	Marker Processing	Preprocessing SNPs	1	00:00:10	00:00:10	00:00:00

**6**	Connected component analysis	Preprocessing traits	1	00:00:56	00:00:56	00:00:00

**7**	Spectral clustering	Preprocessing traits	118	00:00:56	00:06:44	01:43:03

**8**	Trait Processing	Preprocessing traits	1	00:06:02	00:06:02	00:00:00

**9**	GFlasso stage 1	GFlasso optimization	130	00:51:34	01:41:36	110:02:30

**10**	GFlasso validation error stage 1	GFlasso optimization	1	01:43:01	01:43:01	00:00:00

**11**	GFlasso stage 2	GFlasso optimization	130	05:49:08	29:27:45	726:59:05

**12**	GFlasso validation error stage 2	GFlasso optimization	1	00:45:26	00:45:26	00:00:00

**13**	GFlasso stage 3	GFlasso optimization	130	03:18:04	10:56:18	418:12:37

**14**	GFlasso validation error stage 3	GFlasso optimization	1	00:56:32	00:56:32	00:00:00

GFlasso is a structured association mapping algorithm that finds associations between the genome and correlated traits by leveraging the network structure between traits [9]. Auto-SAM uses the proximal-gradient optimization method for GFlasso [13].

The goal we had when we incorporated GFlasso into Auto-SAM was to develop a process that would run GFlasso for as many SNPs and as many traits as possible, while maintaining the integrity of the algorithm. After extensive testing using our test datasets (mouse and yeast), we found that we could run our optimization code for GFlasso on datasets with up to 4000 SNPs and 250 without running out of memory. Auto-SAM supports loading in datasets larger than this, and so we introduced a series of preprocessing steps to prepare for the analysis. These preprocessing steps are performed automatically in Auto-SAM prior to running GFlasso.

Auto-SAM's processing pipeline for GFlasso proceeds as follows: once the analyst starts a GFlasso job in Auto-SAM, the data are downloaded to the cluster from the database and preprocessing starts. The first four preprocessing steps (steps 1-4 in Table 2) are run to find association results via the lasso. First, the lasso is run on the data for each trait, in parallel, for a given *λ *vector, where *λ *is the regularization parameter that controls the level of sparsity. Once all lasso runs complete, the results are combined and the *λ *with the smallest validation error is used to define a new vector for a fine-tuned search. The two steps are subsequently repeated to find the lasso results, which are used in the marker processing step (step 5). These results from the lasso are used to select up to 4000 SNPs to be used to run GFlasso. Because GFlasso looks for SNPs that are associated with correlated traits, we select the SNPs that are associated with the most traits in the lasso results matrix.

The next three steps in the pipeline preprocess the traits. Unlike the SNPs, the traits can be split up into smaller sub-networks and run in parallel, assuming no edges between sub-networks. We find these sub-networks first by finding all connected components (step 6). For all connected components greater than 250 traits, we run spectral clustering [30] to break the sub-network down further (step 7). Once sub-networks have been identified in these steps, the trait processing step (step 8) combines the connected components into sub-networks of 250 traits and GFlasso is then run in parallel on each sub-network.

Three two-step processes run the GFlasso optimization. Auto-SAM spawns ten GFlasso runs for each sub-network of traits, each with a different division of the data (step 9). After all ten runs have finished for all sub-networks, an error calculation step calculates the cross-10-validation error to select the best regularization parameters (*λ *is the regularization parameter that affects the sparsity of the results and *γ *affects the fusion penalty for correlated traits) for that step (step 10). Following a linear search pattern, these two steps are first run for a vector of *λ *given *γ *(step 9 & 10), then for a vector of *γ *given *λ *(step 11 & 12), and finally for *λ *given *γ *(step 13 and 14). Once GFlasso completes, the GFlasso results are inserted into the database, which can then be accessed by the analyst.

Thus, a fourteen-step process is automated in Auto-SAM. This pipeline preprocesses the data, runs GFlasso optimization in parallel, and finds a solution optimal according to cross-ten validation. Whereas the available MATLAB code can only handle datasets with 250 traits, Auto-SAM can run datasets with up to 20,000 traits. We follow a similar process to automate many other structure-finding and structured association mapping algorithms.

To provide quantitative support for the parallelization scheme used in Auto-SAM, we analyzed the running time for each step of the GFlasso process using the yeast data. In Table 2, we report the overall time Auto-SAM spent in each step, the number of parallel jobs that were run, the average time per job, and the total savings due to the parallelization (total compute time for all jobs minus actual real time). We report that overall, six of the jobs run faster with the parallelization, some up to 66 times faster.

### Use case study: running an analysis on simulated data

In this section, we present a short use-case to demonstrate how an analyst can upload data into GenAMap and run structured association analyses. Our intention is that interested readers can follow along with this section to learn how the software works.

In this demonstration, we will use a small simulated dataset. This dataset has 5000 SNPs and 1400 genes for 100 individuals and can be downloaded from the GenaMap downloads page http://sailing.cs.cmu.edu/genamap/join.html. While the size of this dataset is typical to some association mapping datasets, there are also cases where more data is available. Auto-SAM can store datasets of sizes up to 15,000 SNPs for 250 individuals with 20,000 genes. In cases where more SNPs are available than Auto-SAM can handle, analysts can trim their dataset before importing into Auto-SAM. Analysts could select SNPs based on SNP-tagging; alternatively, analysts could select SNPs in known genomic regions of interest or import the top SNPs associated with certain traits using a pairwise association test. On a related note, Auto-SAM has limited support for missing values. Thus, analysts should generally impute missing values before importing data into Auto-SAM.

When the analyst fires up GenAMap for the first time, he/she is presented with three panels - the data panel, algorithm control center, and visualization screen. The analyst interacts with the data panel to create a project and import data. There are three tabs in the data panel representing genetic markers, phenotypes, and associations. The first thing that the analyst needs to do is to right click on the "Projects" label in the data panel and create a new project. Once the analyst has created a new project, marker and trait data can be added to the project.

The example dataset contains four files. *example_gen.txt *is the genotype file, and *example_chrkey.txt *is the key file that describes the location of the SNPs across the genome. The analyst needs both of these files to create a new markerset in a project. To do this, the analyst right clicks on the project that he/she wants to add the data to and selects to *Add Marker Data*. A popup window opens for the analyst to browse to the marker file, which in this case is *example_gen.txt*. The marker file format is strict, with the number of rows representing the number of samples and the number of columns representing the number of SNPs. SNPs can be encoded with any numeric value, although we recommend a 0/1/2 encoding based on the number of minor alleles for the individual at the locus. Before the data can be imported, the analyst must also browse to the SNP key: *example_chrkey.txt*. The sample label file is optional. If it is not used, the samples must be in the same order in the genotype and phenotype files, as is the case in this dataset. The delimiter is set to "w," meaning all white space characters. The analyst can look at other example files formatting by clicking on any of the buttons labeled with a "?" in the import dialog. The analyst must also choose a descriptive name without special characters to describe the markerset. Once the form has been filled out, the analyst selects to import the markers. The markers are imported on a separate thread.

While the markers are loading, the analyst can import the gene values. These values are stored in *example_phen.txt *and *example_phen_key.txt*. The import process for the traits is similar to the marker import process; however, the analyst has a few more options as far as file format goes. To import this dataset, the analyst needs to set the trait file to *example_phen.txt *and the trait label file to *example_phen_key.txt*. The sample label file is not used. The analyst selects *Saccharomyces cerevisiae *as the species and changes the format to *no row or columns headers*. The data can then be imported as before.

The user can use GenAMap to validate the import of the markers and the traits. SNPs can be viewed from the *Marker *tab using GenAMap's genome browser. To view the traits, the analyst must create a network. This is done by right-clicking on the traitset in the *Traits *tab. An algorithm dialog box is presented. The analyst selects the type of network to create, and starts the algorithm. A new job is created in Auto-SAM, and a tracking code is displayed in the algorithm control center. The analyst can right click on the label of the job to stop its execution, find out error information, or restart a job in error. Once the network has been created, the analyst can zoom in and view the genes as described in the GenAMap online tutorials.

The analyst can also add an association set to the project once traits and markers have been added. To do so, the analyst right clicks on the project and selects to add an association. A dialog appears and the analyst can select a traitset. Once a traitset has been selected, all markersets with the same number of samples as the selected traitset are then available for the analyst to choose from. The analyst can then start and monitor association algorithms in the same manner as the network algorithms.

In this use case, we have shown how analysts can import data and run algorithms in the Auto-SAM system. Once the results have completed, they can download the results as text files, or use GenAMap to further explore and analyze the results.

## Discussion and Conclusions

If current trends continue, the amount of data available to biologists will continue to grow at an increasing rate. Biological studies will need to rely on advances in large scale statistical and machine learning more and more as the complexity and amount of data explodes. However, the integration of machine learning advances could become a bottleneck to discovery if the distribution and acceptance of state-of-the-art methods is not improved. In this paper, we have proposed a new deployment strategy that makes the latest machine learning technology in genetics association mapping available to genetics analysts. We have created an automatic processing system called Auto-SAM, which automates four state-of-the-art structured association mapping algorithms. Additionally, we have integrated Auto-SAM into a powerful visualization software system called GenAMap. We have demonstrated that Auto-SAM enables genetics analysts to run a variety of structure and association mapping algorithms without the effort to format the data and customize the implementations. We anticipate that Auto-SAM will enable genetics analysts to incorporate structured association mapping algorithms in their GWAS analysis pipelines, potentially enabling discovery and leading to new genetics insight.

## Availability and requirements

• **Project Name: **GenAMap

• **Project Home Page: **http://sailing.cs.cmu.edu/genamap

• **Operating systems: **Windows, Mac

• **Programming language: **Java

• **Other requirements: **Java 1.6 or higher

• **License: **Non-commercial research use

• **Any restrictions to use by non-academics: **License needed

## Abbreviations

AMTL: Adaptive multi-task lasso; Auto-SAM: Automated structured association mapping system; GFlasso: Graphical-fused lasso; GWAS: Genome wide association studies; MPGL: Multi-population group lasso; SNP: Single nucleotide polymorphism; TreeLasso: Tree-guided group lasso.

## Competing interests

EPX and REC have applied for a provisional patent for the GenAMap visual analytics system, which includes Auto-SAM.

## Authors' contributions

REC and EPX conceived of the software. REC designed the software system and implemented it with help from AG, under the supervision of EPX. REC drafted the manuscript with input from EPX and AG. All authors read and approved the final manuscript.
